# Supplementary material for: FK506-Binding Protein 11 Is a Novel Plasma Cell-Specific Antibody Folding Catalyst with Increased Expression in Idiopathic Pulmonary Fibrosis
Source: Cells. 2022 Apr 14;11(8):1341. doi: 10.3390/cells11081341 (PMC9027113; doi:10.3390/cells11081341)
Supplement: Supplementary file 1 [file cells-11-01341-s001.zip › cells-1604632-supplementary.pdf]

ONLINE DATA SUPPLEMENT

FK506-BINDING PROTEIN 11 IS A NOVEL PLASMA CELL-SPECIFIC ANTIBODY FOLDING CATALYST

Stefan Preisendörfer[1], Yoshihiro Ishikawa[2#], Elisabeth Hennen[1], Stephan Winklmeier[3], Jonas Schupp[4], Larissa Knüppel[1], Isis E. Fernandez[1], Leonhard Binzenhöfer[1], Andrew Flatley[5], Brenda M. Juan-Guardela[4], Clemens Ruppert[6], Andreas Guenther[6], Marion Frankenberger[1], Rudolf Hatz[7,8], Nikolaus Kneidinger[9], Jürgen Behr[9], Regina Feederle[5], Aloys Schepers[5], Anne Hilgendorff[1], Naftali Kaminski[4], Edgar Meinl[3], Hans Peter Bächinger[2], Oliver Eickelberg[1§], and Claudia A. Staab-Weijnitz[1*]

[*]To whom correspondence should be addressed:

Claudia Staab-Weijnitz, Comprehensive Pneumology Center, Ludwig-Maximilians-Universität and Helmholtz Zentrum München, Max-Lebsche-Platz 31, 81377 München, Germany, Tel.: 0049(89)31874681; Fax: 0049(89)31874661; Email: staab-weijnitz@helmholtz-muenchen.de

ORCID-ID: https://orcid.org/0000-0002-1211-7834

Supplementary Methods

Cell culture

A549 (human lung adenocarcinoma cell line) cells were purchased from ATCC (Manassas, VA, USA) and maintained in DMEM/F12 medium (Life Technologies, Carlsbad, CA, USA) supplemented with 10% FBS (Pan Biotech, Aidenbach, Germany) and 100 U/mL penicillin/streptomycin (Life Technologies). Raji (human Burkitt′s lymphoma cell line) cells were a kind gift from Prof. Edgar Meinl (Ludwigs-Maximilians-Universität München) and were cultured in RPMI 1690 medium (Life Technologies) supplemented with L-Glutamine, 10% FBS (Pan Biotech), 10mM Sodium Pyruvate (Life Technologies), Non-essential Amino Acid Solution (1x, Sigma-Aldrich, St. Louis, MI, USA) and 100 U/mL penicillin/ streptomycin (Life Technologies). The mouse myeloma cell line P3X63-Ag8.653 (AG8) and hybridoma cell lines were obtained from the monoclonal antibody core facility (Helmholtz Zentrum München) and cultured under the conditions described for Raji above. Cells were maintained at a density ranging from $2x10^5$ – $9x10^5$ cells/ml. Finally, Peripheral Blood Mononuclear Cells (PBMCs) were isolated from whole blood as shown below and cultured in the same medium as Raji and myeloma/hybridoma cells. All cells were cultivated at 37°C in a direct heat CO^2 incubator (Thermo Fisher Scientific, Waltham, MA, USA) containing 5% CO^2. All experiments were repeated at least three times.

Transfection of A549 cells

For experiments involving A549 cells, knockdown was achieved using reverse transfection. First, transfection mixtures containing Opti-MEM™ I Reduced Serum Medium (Thermo Fisher Scientific), Lipofectamine RNAiMAX (Life Technologies) and relevant siRNA (FKBP11 siRNA s27898 , XBP1 siRNA 5533, both human, Life Technologies) or negative control siRNA No. 1 (AM4611, Life Technologies) were prepared and incubated for 20 minutes in 6 well

plates. Next, A549 cells at 70-80% confluency were detached using trypsin and resuspended in DMEM/F12 medium supplemented with 10% FBS. The resulting cell suspensions were transferred onto the prepared transfection mixtures. The final siRNA concentration was 10 nM, and the cells were seeded at a density of 50.000 – 55.000 cells/cm^2. After an incubation time of 6 – 8 hours, A549 cells were starved overnight using starvation medium (DMEM/F12, 0.5 % FBS) and treated with tunicamycin (Sigma-Aldrich) at indicated concentrations (0.01µg/ml, 0.1µg/ml, 1µg/ml) or equal volume of DMSO as a negative control. After 24 hours, cell viability was assessed by Trypan Blue exclusion assay and protein and RNA was isolated for subsequent Western Blot and qPCR analysis.

Induction of ER stress in Raji cells

To induce ER stress in the human B cell line Raji, cells were washed once in PBS and resuspended in starvation medium (RPMI 1640, 0.5 % FBS) at a density of 3 x 10^5 – 5 x 10^5 cells/ml. Following 24 hours of starvation, cells were washed with PBS and resuspended in starvation medium containing different concentrations of tunicamycin (0.01 mg/ml, 0.1 µg/ml, 0.5 µg/ml, 1 µg/ml or 5 µg/ml) or an equal volume of the solvent DMSO as vehicle control. Expression of FKBP11 was then assessed on both protein and transcript levels. To verify successful induction of ER stress, expression of BiP was visualized using Western Blot analysis.

Isolation, culture and differentiation of PBMCs

Blood was drawn from healthy individuals and left in heparinized vials (Sarstedt AG) for a maximum of one hour at room temperature. Next, blood was mixed in equal parts with PBS and the mixture was carefully poured into a 50ml centrifugation tube containing 15ml of density gradient medium (Lymphoprep, Axis-Shield, Oslo, Norway). The vial was centrifuged for 20min at 1080 x *g* at 20°C without brake, resulting in the PBMCs being gathered in a single

layer. PBMCs were transferred to pre-warmed RPMI medium (Thermo Fisher Scientific) supplemented with 10% FBS, L-glutamine, sodium bicarbonate and non-essential amino acids and once washed. Afterwards, PBMCs were seeded in a cell culture dish at a concentration of $1x10^6$ cells/ml and stimulated by 1000 U/ml of recombinant IL-2 (Roche) and 2.5 μg/ml TLR 7+8 ligand R848 (InvivoGen, San Diego, CA, USA. As a control, same volumes of diluents were added to PBMCs. After 7 days of incubation at 37°C cytospins were prepared, and RNA and protein was extracted from remaining cells.

Trypan Blue exclusion assay

To estimate the number of viable cells, cell suspensions were mixed with equal volumes of Trypan Blue solution (1:10 in PBS, Sigma-Aldrich) and viable cells counted in a Neubauer chamber (Celeromics, Grenoble, France).

RNA isolation and Real-Time quantitative Reverse-Transcriptase PCR (qRT-PCR) analysis

For isolation of RNA from cell cultures the RNeasy Mini Kit (QIAGEN, Hilden, Germany) or the total RNA kit peqGOLD (Peqlab, Erlangen, Germany) was used. For suspension cell cultures, cells were spun down at 300 x *g* and the pellet was resuspended in the lysis buffer provided with the kit. Then, the instructions provided with the kit were followed. For adherent cells, instead of centrifugation, the cells were detached using a cell scraper.

RNA was reverse transcribed in a total volume of 40 μl according to the manufacturer's protocol (Life Technologies), including Reverse Transcriptase (Applied Biosystems, Foster City, CA, USA). Finally, to determine relative transcript abundance of a specific gene, quantitative real-time PCR (qRT-PCR) was performed using SYBR Green PCR master mix (Roche Diagnostics GmbH, Mannheim, Germany) and primer mixtures shown in supplementary Table S1. The reaction procedure was as follows: 95°C for 5 min, 45 cycles of 95°C for 5min, 59°C for 5 sec and 72°C for 10 sec. Results were indicated as "$-\Delta C_t$" values

($-\Delta C_t = C_t^{reference} - C_t^{target}$). For standardization of relative mRNA expression, GAPDH was employed as endogenous control.

Protein isolation and Western Blot analysis

To isolate protein from liquid nitrogen-frozen tissue, samples were homogenized using a micro-dismembrator (Sartorius, Göttingen, Germany) and taken up in Radio-Immunoprecipitation Assay (RIPA) buffer (50 mM Tris-HCl pH 7.4, 150 mM NaCl, 1 % Triton X100, 0.5 % sodium deoxycholate, 1 mM EDTA, 0.1 % SDS) containing a protease and a phosphatase inhibitor cocktail (Roche). The solution was then incubated for 30 min on ice, succeeded by short sonification and centrifugation for 15 min at 13.000 rpm at 4°C to clarify the lysates. Pellets were discarded and protein concentration was measured via Pierce BCA Protein Assay (Thermo Fisher Scientific).

To isolate protein from cultured cells, suspension cells were spun down at 300 x *g* and pellets were incubated in RIPA buffer supplemented with a protease and a phosphatase inhibitor cocktail for at least 30 minutes on ice, followed by centrifugation at 15.000 rpm at 4°C. The pellets were discarded and protein concentration was determined as described above. For adherent cells, cells were detached using cell scrapers.

After denaturation of protein samples by Laemmli buffer (65 mM Tris-HCl pH 6.8, 10 % Glycerol, 2 % SDS, 0.01 % bromophenolblue, 100 mM DTT) at 95°C for 5 min, proteins were separated via SDS-PAGE and blotted to a polyvinylidene difluoride (PVDF) membrane. Membranes were blocked with 5 % milk in TBS-T (0.1% Tween 20, TBS), followed by overnight incubation with primary antibodies (see supplementary Table S2) at 4°C. After washing the membrane in TBS-T for three times, the membrane was incubated with an appropriate secondary antibody (1:20.000, all GE Healthcare Life Sciences, Freiburg, Germany) for 1 h at room temperature. Again, the membrane was washed three times, and

proteins were visualized with the ChemiDocXRS+ imaging system (Bio-Rad, München, Germany) using a suitable substrate (SuperSignal^{TM} West Dura Extended Duration Substrate or SuperSignal^{TM} West Femto Maximum Sensitivity Substrate, Thermo Fisher Scientific). Detected bands were quantified using Image Lab software (Bio-Rad, Hercules, CA, USA), and relative protein abundance was calculated by dividing the band intensity of the target protein with the band intensity of the loading control (ACTB) of the same sample.

Immunofluorescent staining of tissue sections

For deparaffinization and rehydration, paraffin-embedded sections were placed at 60°C for at least 30 min, followed by incubation in xylene (2 times for 5 min) and immersion in a descending alcohol series (100% EtOH for 2min, 100% EtOH for 2min, 90% EtOH for 1min, 80% for 1min, 70% for 1min) at room temperature. After rinsing the sections in deionized water, slides were transferred into citrate buffer (pH 6.0) and heated in a Decloaking Chamber for 30 seconds at 125°C, and 10 seconds at 90°C, in order to retrieve antigens. Subsequently, sections were allowed to cool down and placed into a blocking solution consisting of 5% BSA in Tris buffer (0.5M Tris, 1.5M NaCl, pH 6.8) for 40 min to prevent nonspecific binding. Next, primary antibody solutions (table 2) prepared in antibody diluent (Zytomed Systems, Berlin, Germany) were applied to each tissue section for 1 hour and washed three times in Tris buffer (5 min for each washing step). Slides were then incubated with secondary antibodies conjugated to appropriate fluorophores (1:250, all Life Technologies) and DAPI (1:1000, Sigma-Aldrich) for 1 hour. Again, slides were rinsed three times in Tris buffer and covered with Flourescence Mounting Medium (Dako, Hamburg, Germany). For examination of the stainings, an Axio Imager Microscope (Carl Zeiss, Jena, Germany) was used.

To quantify $FKBP11^+/CD38^+$ cells in IPF and donor sections, ten images sized $1.5mm^2$ were randomly taken of each section. Then, $FKBP11^+/CD38^+$ cells were counted from each image, and the counted cells for all 10 images were added up. The observer was blinded to diagnosis.

Immunoflourescent staining of cytospins

For generation of cytospins, 100 µl of cell suspension (0.8 – 1.2 x 10^6 cells/ml) was transferred into a cytofunnel and spun down to a slide at 300 rpm using a Cytospin™ 4 Cytocentrifuge (Thermo Fisher Scientific). All following steps were performed at room temperature. The cytospins were allowed to dry overnight and the cells were fixed by application of 3.7% paraformaldehyde (PFA) in DPBS for 5 min. After washing once with DPBS, cells were permeabilized with 0.2% Triton X-100 in DPBS for 2 min. Next, cells were washed once in DPBS and blocking was performed with 5% BSA, 0.2% Tween, DPBS. Primary antibodies prepared in blocking solution were then applied onto the cells for 1 hour, followed by washing three times in DPBS. Subsequently, secondary antibodies conjugated to appropriate fluorophores (1:250, all Life Technologies) and DAPI (1:1000, Sigma-Aldrich) were incubated for 1 hour. Finally, the cytospins were washed three times with DPBS and covered with Flourescence Mounting Medium (Dako). Stainings were inspected using an Axio Imager Microscope (Carl Zeiss).

SUPPLEMENTARY TABLES

Table S1. Primer table for qRT-PCR. Primers were synthesized by MWG Eurofins (Ebersberg, Germany).

Target	Species	NCBI accession numbers of targeted transcripts	Forward primer (5'-3')	Reverse primer (5'-3')
FKBP10	human	NM_021939.4	CGACACCAGCTACAGTAAG	TAATCTTCCTTCTCTCTCCA
FKBP11	human	NM_016594.3<br>NM_001143781.2	GCAATCATTCCTTCTCACT	AGTAGTTGGCTCGGATTAG
PRDM1	human	NM_001198.4<br>NM_182907.3	GGAACTTCTTGTGTGGTATT	TCTGTGTTTGTGTGAGATTC
XBP1	human	NM_005080.4<br>NM_001079539.2<br>NM_001393999.1<br>NM_001394000.1	CTGAGTCCGCAGCAG	TCCAAGTTGTCCAGAATG
GAPDH	human	NM_002046.7<br>NM_001256799.3<br>NM_001289745.3<br>NM_001289746.2	TGACCTCAACTACATGGTTTA<br><br>CATG	TTGATTTTGGAGGGATCTCG
HPRT	human	NM_000194.3	AAGGACCCCACGAAGTGTTG	GGCTTTGTATTTTGCTTTTCCA

Table S2. Primary antibodies. Primary antibodies used for Western Blot analysis and immunofluorescence stainings.

Target	Antibody	Provider	Application
ACTB	HRP-conjugated anti-ACTB antibody	Sigma Aldrich, St. Louis, USA	WB
α-SMA	mouse monoclonal anti-ACTA2	Sigma Aldrich, St. Louis, USA	WB
BiP	rabbit monoclonal anti-BiP	Cell Signaling, Boston, USA	WB
Calreticulin	rabbit polyclonal anti-Calreticulin antibody	Cell Signaling, Boston, USA	WB
CD27	mouse monoclonal anti-CD27	Abcam, Cambridge, United Kingdom	IF
CD38	mouse monoclonal anti-CD38	Santa Cruz, Dallas, USA	IF
CD45	mouse monoclonal anti-CD45	Sigma-Aldrich, St. Louis, USA	IF
CD138	mouse monoclonal anti-CD138	Sigma-Aldrich, St. Louis, USA	IF
FKBP5	rabbit monoclonal anti-FKBP5	Cell Signaling, Boston, USA	WB

FKBP11	rabbit polyclonal anti-FKBP11	ATLAS, Stockholm, Sweden	WB, IF
GAPDH	HRP-conjugated anti-GAPDH antibody	Cell Signaling, Boston, USA	WB
IgA	goat polyclonal anti human IgA	Sigma-Aldrich, St. Louis, USA	WB, IF
IgG	rabbit monoclonal anti human IgG	Abcam, Cambridge, United Kingdom	WB
IgG	mouse monoclonal anti human IgG	Abcam, Cambridge, United Kingdom	IF
IgM	goat polyclonal anti human IgM	Sigma-Aldrich, St. Louis, USA	WB, IF
Lamin A/C	rabbit polyclonal anti-Lamin A/C antibody	Cell Signaling, Boston, USA	WB
PDIA3	mouse monoclonal anti-ERp57 (PDIA3)	Abcam, Cambridge, United Kingdom	WB

Table S3. Antibodies used for Flow Cytometry. All antibodies were purchased from BioLegend, San Diego, CA, USA

**Antigen**	**Fluorophore**	**Clone**	**Isotype**
CD3	APC/Cy7	HIT3a	mouse IgG2a, κ
CD20	APC/Cy7	2H7	mouse IgG2b, κ
CD27	PE/Cy7	O323	mouse IgG1, κ
CD38	APC	HB-7	mouse IgG1, κ

SUPPLEMENTARY FIGURES

Figure S1: Protein levels of FKBP5 are not altered in IPF. Western Blot analysis of protein extracts from normal histology control (n=5) and IPF lungs (n=6). In contrast to transcript levels, FKBP5 protein levels, as assessed by Western Blot analysis, were not decreased.

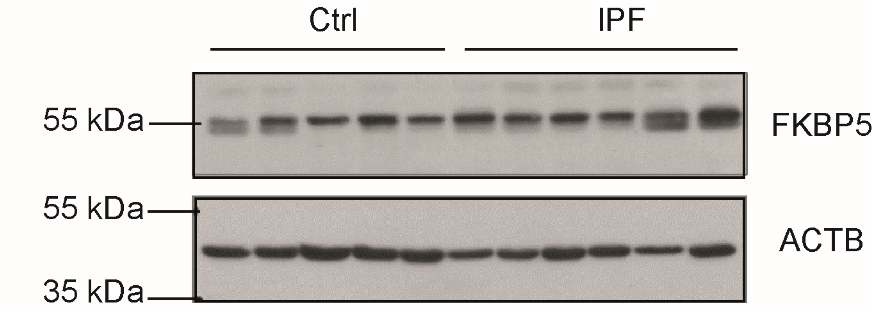

Figure S2: Correlation of *FKBP11* expression with demographic and clinical parameters. *FKBP11* transcript abundance values (log2 values) extracted from microarray data of samples from patients with IPF (*n* = 99) were correlated with (**A**) forced vital capacity (FVC, % of predicted), (**B**) diffusing capacity for carbon monoxide (DLCO), (**C**) age, (**D-F**) quality-of-life scores including (**D**) the St. George's Respiratory Questionnaire (SGRQ) score, (**E**) the Short Form (12) Health Survey for general health (SF-12 PCS) and (**F**) for mental health (SF-12 MCS), (**G**) sex, (**H**) 6-minute walk test (6MWT), (**I**) smoking status, and (**J**) pack years. All data is from the National Lung Heart and Blood Institute Lung Tissue Research Consortium (NLHBI LTRC) [1, 2].

Figure S2

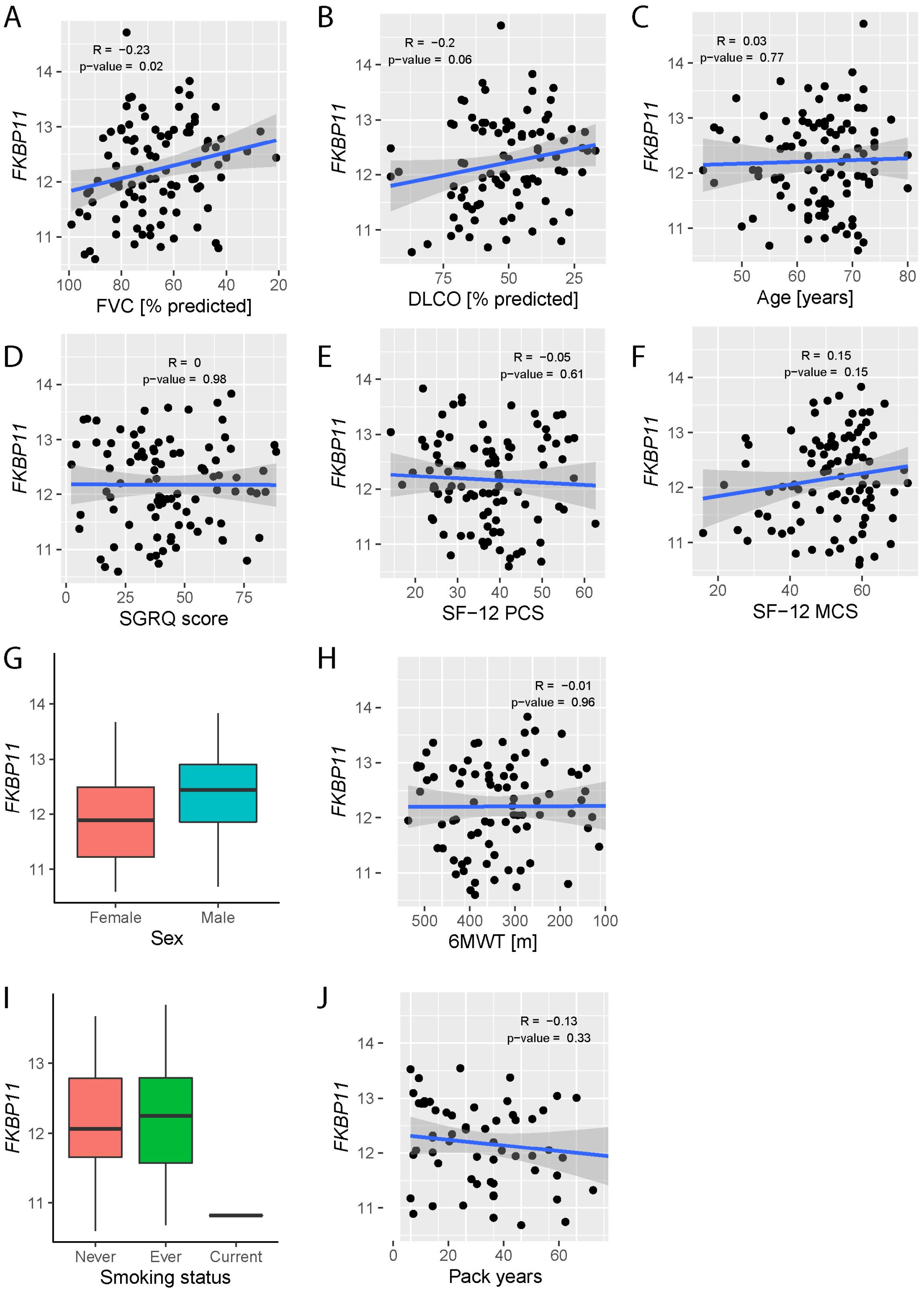
A
R = −0.23
p−value = 0.02
FKBP11
FVC [% predicted]
B
R = −0.2
p−value = 0.06
FKBP11
DLCO [% predicted]
C
R = 0.03
p−value = 0.77
FKBP11
Age [years]
D
R = 0
p−value = 0.98
FKBP11
SGRQ score
E
R = −0.05
p−value = 0.61
FKBP11
SF−12 PCS
F
R = 0.15
p−value = 0.15
FKBP11
SF−12 MCS
G
FKBP11
Female
Male
Sex
H
R = −0.01
p−value = 0.96
FKBP11
6MWT [m]
I
FKBP11
Never
Ever
Current
Smoking status
J
R = −0.13
p−value = 0.33
FKBP11
Pack years

Figure S3: FKBP11 localizes to the ER in primary human lung fibroblasts, but *FKBP11* shows much lower expression than FKBP10, and FKBP11 deficiency does not affect myofibroblast differentiation and collagen secretion. (**A**) Western blot analysis of subcellular fractionation of primary human lung fibroblasts. FKBP11 is enriched in the microsomal extract (ME), similar to the ER-resident proteins calreticulin (CALR) and protein disulfide isomerase A3 (PDIA3). Also similar to both ER-resident proteins, a smaller amount of FKBP11 is additionally found in the nuclear extract (NE) but very little in the chromatin-bound fraction (CB) and the cytosolic extract (CE). Lamin A/C and glyceraldehyde phosphate dehydrogenase (GAPDH) were used as marker proteins for NE/CB and CE, respectively. (**B**) Baseline expression of *FKBP11* in comparison to *FKBP10* in primary human lung fibroblasts as determined by qRT-PCR analysis. On average, *FKBP10* expression is more than 4-fold higher than *FKBP11* expression. (**C**) Representative Western Blot showing that FKBP11 knockdown in primary human lung fibroblasts does not affect myofibroblast differentiation as assessed by levels of α-smooth muscle actin (ACTA2). (**D**) Results of Sircol assay demonstrating that FKBP11 does not significantly affect collagen secretion by primary human lung fibroblasts. Sircol assay was carried out as described in Staab-Weijnitz *et al* [3].

Figure S3

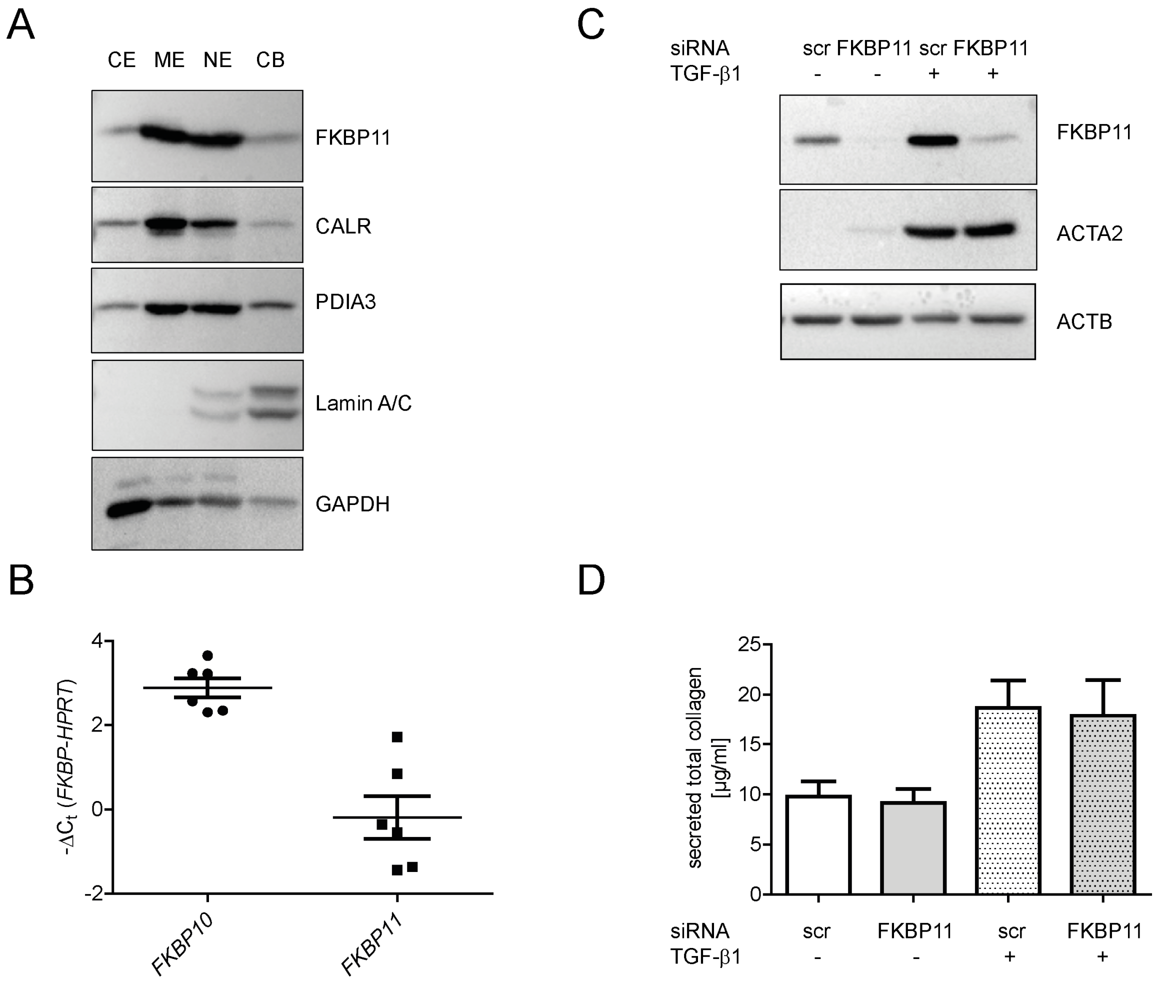
A
CE ME NE CB
FKBP11
CALR
PDIA3
Lamin A/C
GAPDH
B
-ΔCt (FKBP-HPRT)
4
2
0
-2
FKBP10
FKBP11
C
siRNA scr FKBP11 scr FKBP11
TGF-β1 - - + +
FKBP11
ACTA2
ACTB
D
secreted total collagen [µg/ml]
25
20
15
10
5
0
siRNA scr FKBP11 scr FKBP11
TGF-β1 - - + +

Figure S4: Correlation of *FKBP11* expression with *MZB1*, *ACTA2*, *COL1A2*, and *COL3A1* expression. *FKBP11* transcript abundance values (log2 values) extracted from microarray data of samples from patients with IPF (*n* = 99) were correlated with (**A**) *MZB1* transcript levels, (**B**) *ACTA2* transcript levels, (**C**) *COL1A2* transcript levels, and (**D**) *COL3A1* transcript levels. All data is from the National Lung Heart and Blood Institute Lung Tissue Research Consortium (NLHBI LTRC) [1, 2]

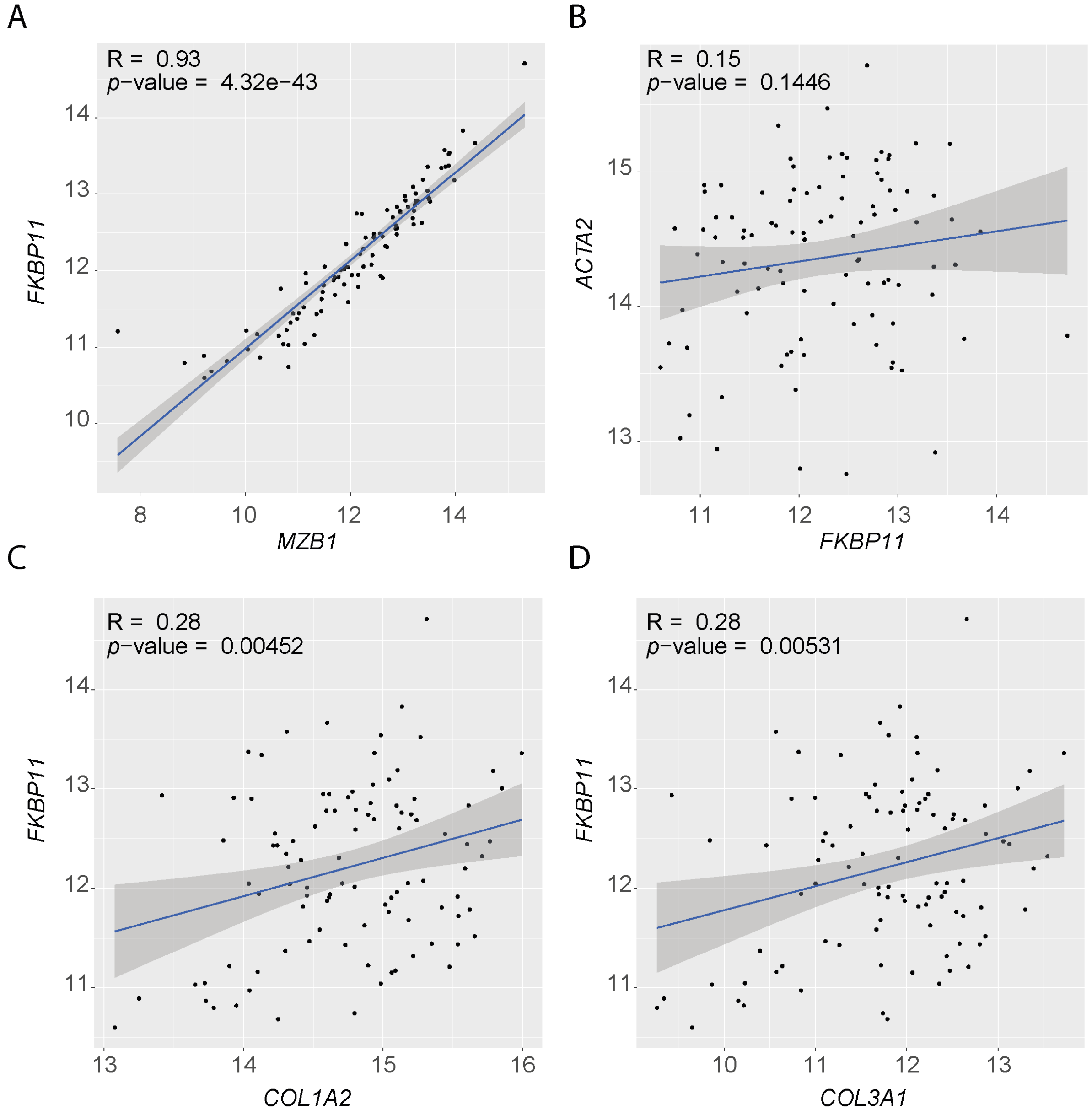

Figure S5: Gating strategy for quantification of plasma cells in peripheral blood of IPF patients in comparison to healthy control. Plasma cells from whole blood of IPF patients and healthy donors were sorted. The double negative population for CD20 and CD3 was analyzed further for expression of CD27 and CD38. Peripheral plasma cells were identified as CD20^{-}/CD3^{-}/CD27^{+}/CD38^{+} cells.

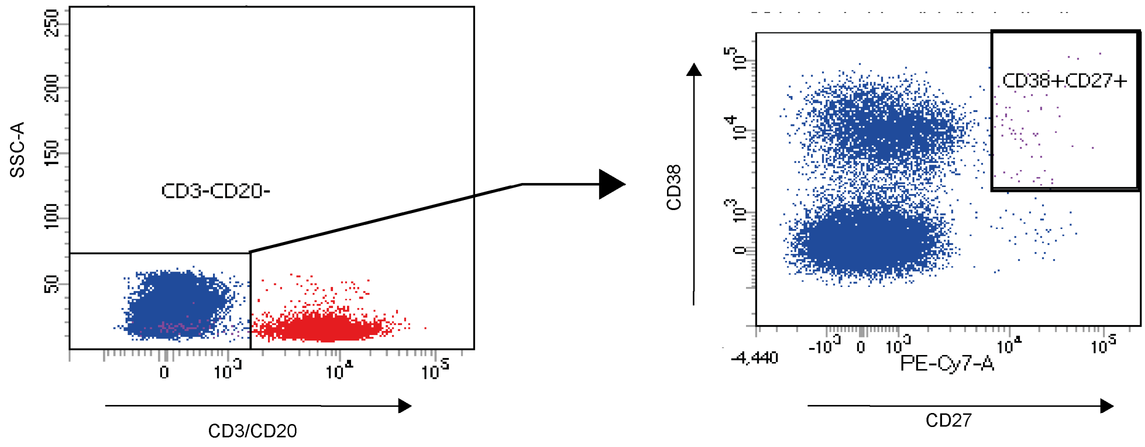

Figure S6: FKBP11 is also detected in non-plasma ($CD38^-$) cells in pancreas and stomach, but not in other healthy human tissue like muscle and lung. Immunofluorescent staining of a human tissue array for FKBP11 and CD38 demonstrated that FKBP11 was also expressed in cells negative for CD38, namely in pancreas and stomach. Other healthy human control tissue like muscle and lung did not show FKBP11 expression. Scalebar 20 µm.

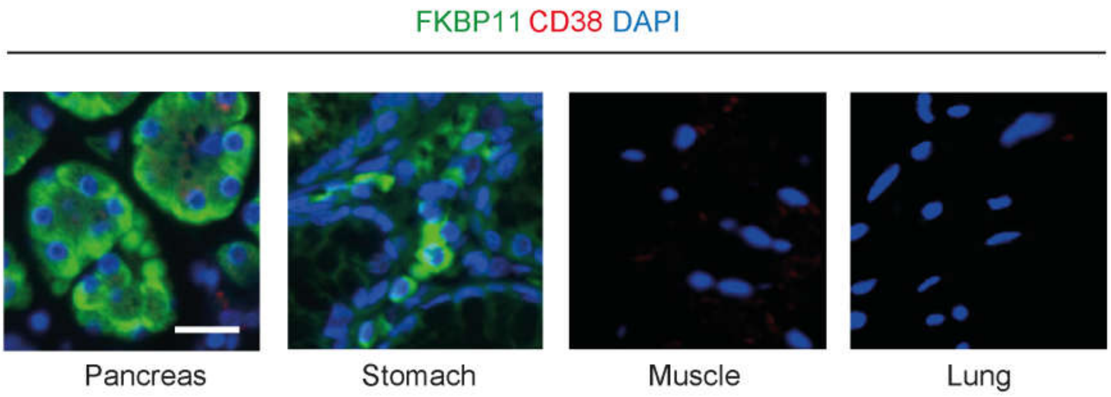

Figure S7: Single cell-RNA-Seq analysis of mouse lungs confirms high plasma cell specificity for *Fkbp11*. Dot blots for *Fkbp11* in comparison to the plasma cell-specific protein *Mzb1* [4] extracted from Angelidis *et al* [5] demonstrate high plasma cell specificity for *Fkbp11 expression*, similar to *Mzb1*. In contrast, the widely accepted antibody foldase cyclophilin B (*Ppib*) as well as *Fkbp2* (also called Fkbp13) are expressed at high abundance by many cell types. Data was obtained via the interactive web tool found on https://theislab.github.io/LungAgingAtlas/.

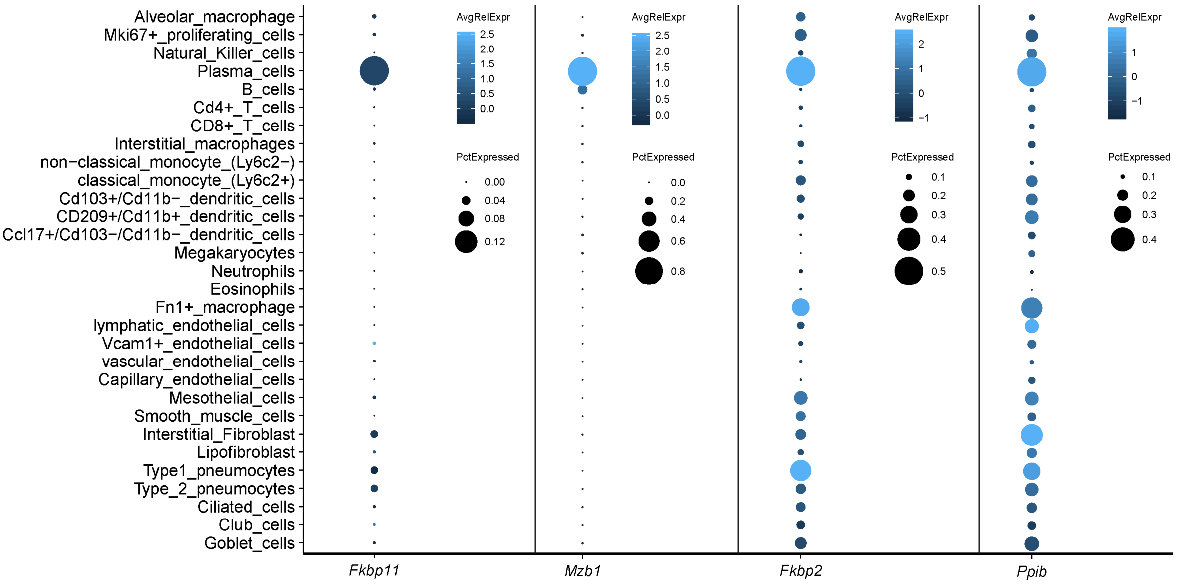

Figure S8: FKBP11 localizes to the ER in A549 and Raji cells and is induced by the UPR also in Raji cells. (**A**) Western blot analysis of subcellular fractionation of A549 (lung adenocarcinoma) and Raji cells (Burkitt's lymphoma cell line), both treated with 0.1 µg/ml tunicamycin. In both cell types, FKBP11 is enriched in the microsomal extract (ME), similar to the ER-resident protein PDIA3. Also similar to PDIA3, a smaller amount of FKBP11 was additionally found in the nuclear extract (NE) but not in the chromatin-bound fraction (CB). Lamin A/C and glyceraldehyde phosphate dehydrogenase (GAPDH) were used as marker proteins for NE/CB and CE, respectively. (**B, C**) Similar to treatment of A549 (*cf.* Figure 4A, B), treatment of Raji cells with the synthetic ER stress inducer tunicamycin led to an increase of *FKBP11* expression both on transcript (**B**) and on protein level (**C**). Upregulation of the ER chaperone HSPA5 (also BiP, GRP78) confirmed induction of ER stress. ACTB = β-actin as loading control.

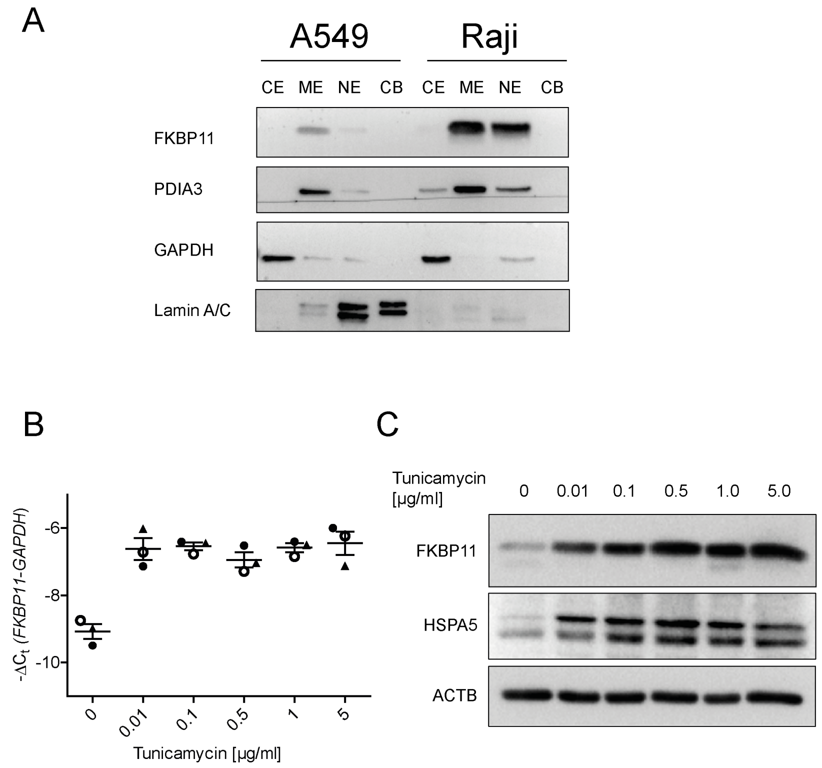

Figure S9: FKBP11 knockdown affects neither IgG HC transcription, nor IgG levels in the ER, nor antibody binding affinity in the assessed hybridoma cell line H3. (**A)** Fold change of IgG heavy chain transcription in response to FKBP11 (n = 6) and PPIB (n = 3) knockdown relative to scr siRNA control. **(B**) Western blot analysis (representative of n = 3, see quantification in (**C**)) of subcellular fractionation of the hybridoma cell line H3 following FKBP11 knockdown. Similar to Supplementary Figures S1 and S6, FKBP11 and all other known ER-resident proteins including HSPA5 and IgG are enriched in the microsomal extract (ME) and to a lesser extent in the nuclear extract (NE). FKBP11 knockdown is visible in both fractions. Lamin A/C and glyceraldehyde phosphate dehydrogenase (GAPDH) were used as marker proteins for NE/chromatin-bound fraction (CB) and cytosolic extract (CE), respectively. (**C**) Quantification of band intensities demonstrates that FKBP11 knockdown did not induce an enrichment of IgG in the ER. Based on the assumption that the bands in NE represent mainly contaminations from the microsomal fraction, bands from both ME and NE were quantified and summed up, but quantifying only the ME-enriched bands yielded very similar results. Results are based on n = 3 and presented as mean ± SEM. (**D, E**) ELISA-based assessment of antibody functionality. After transfection with scr siRNA, FKBP11 siRNA, or PPIB siRNA, hybridoma supernatants showed no difference in binding to the cognate antigen, neither the GST-tagged antigen (**D**) nor the untagged antigen (**E**).

Figure S9

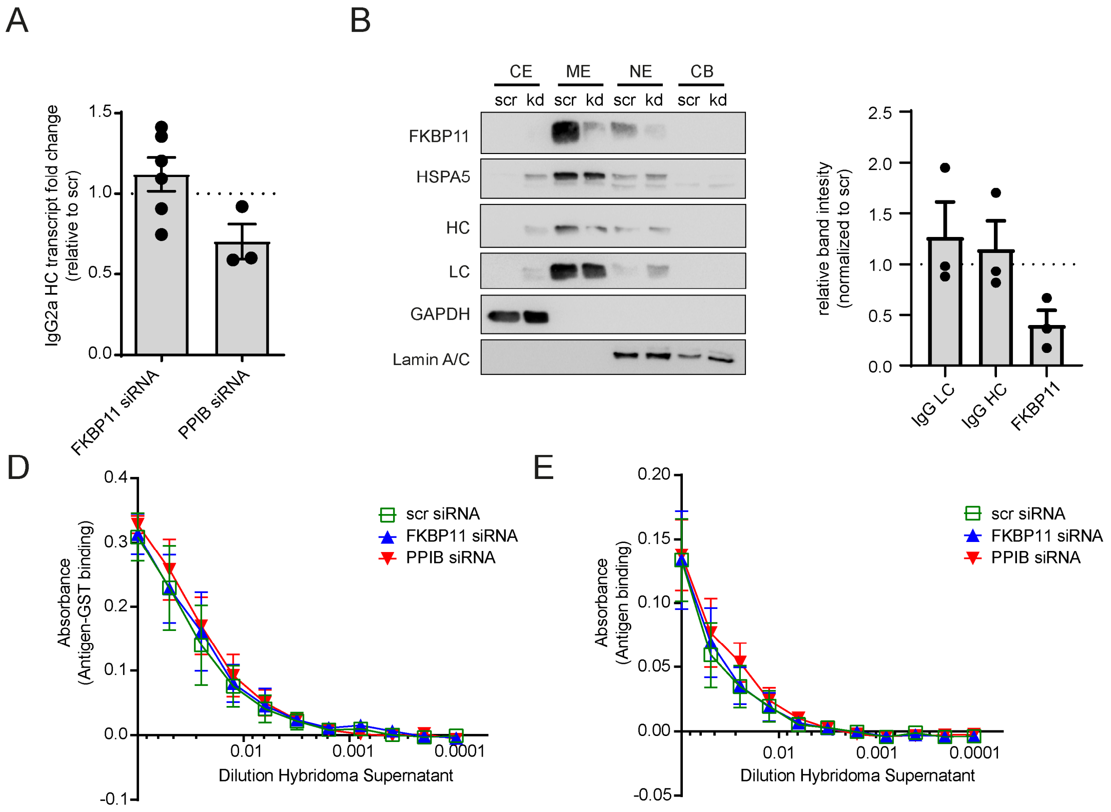

SUPPLEMENTARY REFERENCES

1. Bauer, Y., et al., *A novel genomic signature with translational significance for human idiopathic pulmonary fibrosis.* American Journal of Respiratory Cell and Molecular Biology, 2015. **52**(2): p. 217-31.
2. Yang, I.V., et al., *Relationship of DNA methylation and gene expression in idiopathic pulmonary fibrosis.* American Journal of Respiratory and Critical Care Medicine, 2014. **190**(11): p. 1263-72.
3. Staab-Weijnitz, C.A., et al., *FK506-Binding Protein 10, a Potential Novel Drug Target for Idiopathic Pulmonary Fibrosis.* American Journal of Respiratory and Critical Care Medicine, 2015. **192**(4): p. 455-467.
4. Schiller, H.B., et al., *Deep Proteome Profiling Reveals Common Prevalence of MZB1-Positive Plasma B Cells in Human Lung and Skin Fibrosis.* American Journal of Respiratory and Critical Care Medicine, 2017. **196**(10): p. 1298-1310.
5. Angelidis, I., et al., *An atlas of the aging lung mapped by single cell transcriptomics and deep tissue proteomics.* Nature Communications, 2019. **10**(1): p. 963.
